# The potential of incorporating norm-critical design objects as a pedagogical tool in sociology courses

**DOI:** 10.3389/fsoc.2024.1405374

**Published:** 2024-06-28

**Authors:** Anna Isaksson

**Affiliations:** School of Health and Welfare, Halmstad University, Halmstad, Sweden

**Keywords:** norm-critical design, pedagogical tool, gender, sociology courses, teaching

## Abstract

Previous research has raised challenges in teaching gender theory in sociology courses. While many students appreciate such theories, some students resist sociological approaches to gender, sexualities, and social inequalities. There is a growing body of research that has recognized and explored pedagogical tools aiming to help students engage with sociological insights and concepts related to gender. However, more studies and pedagogical frameworks are needed to guide higher education teachers. Consequently, this perspective article aims to introduce and present how norm-critical design objects can be used as a pedagogical tool to enhance student learning and engagement. The article demonstrates how such objects have been incorporated into sociology courses and provides a springboard for reflective, thorough, and problematizing approaches to gender issues in sociology. Further, the article encourages a broader sociological discussion about the potential of using norm-critical design objects in sociology courses.

## 1 Introduction

As a sociology teacher with 20 years of teaching experience, applying different pedagogical tools to prompt student engagement and learning is still inspiring. In sociology, I have primarily taught about gender and diversity issues, feminist theory, and (the absence of) women in classical sociological theory. I have experienced challenges raised in previous research on teaching gender theory in sociology courses. While many students appreciate these theories and perspectives, some students resist sociological approaches to gender and openly resist the course material (c.f. Miller and Chamberlin, 2000; Horwath and Diabl, 2019).

Some students find concepts such as gender as a social construction challenging to grasp. Students also perceive gender theory (and sociological theory in general) as abstract and disconnected from their everyday lives and future work (Berkowitz et al., 2010; McCabe, 2013). Further, previous studies have demonstrated how teaching gender could be regarded as a “high-risk occupational strategy because feminism and gender studies are constantly contested (…) and challenged by the rise of ‘antigenderism’,” where gender studies are regarded as constituting “a scientific hoax” because sex differences are considered natural and invariable (…) (Heijstra and Pétursdóttir, 2023, 349). Research has demonstrated that students resist validating inequality as a structural phenomenon and even perceive inequality issues as being resolved (Titus, 2010). Another issue raised by Trumpy (2023) concerns that students’ understanding of sex, sexualities, and gender often remains simplistic. Integrating these issues and perspectives throughout the sociological curriculum in a way that deepens students’ understandings can be a challenge.

Consequently, previous studies have highlighted a need for pedagogical frameworks to guide higher education teachers in teaching gender, sexualities, social inequalities, *et cetera*. While some studies suggest that education in general around sexual and gender identities and diversities is characterized by a rather traditional lecturing procedure, with the teacher/professor sharing the content with the students (c.f. López-Orozco et al., 2022), there is a growing body of research that has recognized and explored more innovative pedagogical tools aiming to help students engage with sociological insights and concepts related to gender, sexualities, and social inequalities. Hartless (2021) demonstrates how horror can be used as a pedagogical tool for teaching sexualities. By showcasing societal views and the marginalization of LGBTQ+ individuals, the horror genre aids students in grasping the concept of sexuality as a socially constructed and historically influenced phenomenon, thereby addressing the educational challenges present. However, as argued by Hartless (2021, 239):

(…), teaching sexualities involves not only helping students understand and historicize the complex identities under the LGBTQ+ umbrella but also showing them that heterosexuals also possess sexual identities. For example, horror films can illustrate how gender norms create toxic different-gender relationships where neither participant receives the support they need. Films like May (2020), Teeth (2007), and The Loved Ones (2009) critique how girls are socialized into chaste ideals of heterosexual romance, showing the madness that can result when a “prince” does not come or when he cares more about his own sexual gratification than a woman’s heart and bodily autonomy (…). In addition to illustrating gender norms, the horror genre sends messages about which forms of sexual expression are tolerated in society. Films like Scream (1996) and The Final Girls (2015) self-referentially deconstruct and challenge the conservative morals that have long defined the slasher subgenre, including taboos against premarital sex. Franchises like Hellraiser (1987) address the consequences of sexual practices that are even more stigmatized in society, such as infidelity and BDSM.

In sum, horror has the potential to overcome challenges such as teaching sexualities to students who are antagonistic to the LGBTQ+ community as well as those who are more accepting of students (Hartless, 2021).

Lampe (2023) suggests that the social media app TikTok can be a valuable tool for increasing student engagement in online sociology courses. She utilized TikTok in two online Sociology of Sex and Gender courses at the University of South Carolina to examine how students engage with course content. Her study indicates that utilizing social media within sociology courses that deal with various systems of inequalities could be an effective approach to involving students and enhancing inclusive teaching practices. According to the students, the teaching with TikTok also promoted empathy for other people with different experiences than their own. Further, students emphasized that TikTok helped to simplify big ideas that were perceived as complicated when reading about them. In other words, TikTok allowed them to “simplify” course content that was difficult to grasp by only reading course literature.

When teaching about gender and sexuality, Vaughn and Leon (2021) argue that digital storytelling can be used as a pedagogical tool. Digital storytelling combines visual and auditory media to enhance the narrative, enabling authors to create comprehensive stories that can reach diverse audiences across various disciplines. In gender and sexuality studies, it allows students to explore and describe systems of power and social intersections. Vaughn and Leon further suggest that digital storytelling helps students to apply theoretical frameworks and understand their experiences and identities without marginalizing others. In sum, digital storytelling,

(…) highlight the importance of integrating theory, particularly feminist and black feminist theory, into the discussion and learning of sexuality, help students by immersing them in the sociological world and its theories, teach students valuable technological skills and tricks that they can employ in other settings, and act as a guiding principle when students analyze sexuality and why exactly they behave as they do (Vaughn and Leon, 2021, 254).

Horror, TikTok, and digital storytelling are three very different examples of pedagogical tools. However, they are all interested in enhancing student engagement and learning related to gender, sexuality, and social inequality in sociology courses. This perspective article also shares this interest and aims to introduce another pedagogical tool—norm-critical design objects. Further, the aim is to encourage a sociological discussion about the potential of using norm-critical design objects as a pedagogical tool to help students engage in sociology courses concerning issues like gender, sexuality, and social inequalities.

The article is structured as follows. First, I briefly describe the role and power of design in society to contextualize the norm-critical design approach my research colleagues and I have developed in various research projects to communicate results and engage different stakeholders. Two examples of norm-critical design objects are then presented. This section is followed by a description of how I have incorporated these objects into sociology courses. In conclusion, I raise some relevant questions to discuss and explore further from a sociological perspective.

## 2 The role and power of design in society

In professional design circles, the concept of design has often been referred to as a process of envisioning and creating objects, services, and experiences that meet human needs and enhance people’s lives (Binder et al., 2011). However, many scholars have elaborated on the role of design and argue that design plays a more profound role in our society. Design is shaped by and shapes our societies. Design influences, defines, produces, and transforms people’s interactions with products, services, environments, cultures, and social norms (Liu, 2024). Costanza-Chock (2020) acknowledges the transformation potential of design and describes design as a way of thinking, learning, and engaging with the world. Critical design (Bardzell et al., 2012; Bardzell and Bardzell, 2013; Dunne and Raby, 2013; Malpass, 2013), speculative design (Dunne and Raby, 2013; Wilkie et al., 2015; Michael, 2016), and reflective design (Sengers et al., 2005; Gaver et al., 2013) are, for instance, examples of design research approaches engaging with the world and are typically used to challenge norms and assumptions, provoke thoughts and discussions, address complex problems, explore future scenarios, and inspire change. Aligning with the emerging field of design justice (Costanza-Chock, 2020), these approaches go beyond calls for design for good, user-centered design, and greater diversity in the fields of technology and design. Rather than solving a problem, design is seen as a medium to stimulate discussion and reflection among researchers, designers, and the public (Dunne and Raby, 2013). Further, reasoning through design is regarded as a mode of knowledge production, and design is linked to broader efforts such as social, economic, and ecological sustainability (Costanza-Chock, 2020).

The power of design and design’s role in contributing to or hindering an equitable, just, and sustainable future has been further recognized in various studies. Kaygan et al. (2019) and Ehrnberger et al. (2012) demonstrate how artifacts in our society are gendered and thus risk reinforcing gender bias and stereotypes. Consequently, several scholars have emphasized the urgent need to incorporate gender perspectives in technology, engineering, and design education to foster sustainable cultural transformation. To tackle complex issues, students require support in developing norm-critical gender lenses (González-González et al., 2020; Yetiş and Bakırlıoğlu, 2024). Denz and Eggink (2019) also highlight the significance and urgency of an active “pedagogical push.” They argue that there is a need to prepare and equip future designers to design for political change.

The emerging attention to design as a powerful tool for social, cultural and political change is also reflected in the growing interest in norm-critical design (Fuenfschilling et al., 2022). Norm-critical design acknowledges the notion of design as a mode of knowledge production and has many similarities with critical design, speculative design, and reflective design since norm-critical design is not interested in developing a new product and/or solve a problem. Through norm-critical design, constraining norms are identified and visualized. Discourses, assumptions, and norms we take for granted are called into question. New ideas, critical reflections, and problems are raised, which challenge status quo and the domination of specific ideas, perceptions and norms (Börjesson et al., 2016; Ehrnberger et al., 2017). The following section describes how my colleagues and I have adopted and practiced a norm-critical design approach.

## 3 Norm-critical design objects—examples from previous research

In previous research projects, my colleagues and I have explored and visualized constraining norms in different areas, such as the male-dominated fire and rescue service (Isaksson et al., 2017) and the female-dominated elderly care (Andersson et al., 2019). Based on the results of studies conducted by other researchers (Wickström and Zeiler, 2021), we have also visualized what the Health Behavior in School-aged Children (HBSC) survey study does and means for young people and problematized the performativity of surveys. My colleagues developed a norm-critical design object about 10 years ago—the Androchair (Ehrnberger et al., 2017). I was then involved in follow-up research projects related to the Androchair (Börjesson et al., 2016).

In most of our previous projects, researchers with backgrounds in design, architecture, gender studies, political science, and sociology collaborated and contributed equally to the project outcomes. In several of the projects, the development and utilization of norm-critical design objects was a part of the organization’s innovation efforts and/or gender equality and diversity work. We have typically followed four steps and phases (mapping, analyzing, visualizing, and discussion and reflection) when developing the norm-critical design objects. In the first phase (mapping), we consider previous research, reports, and media and social media debates relevant to the topic. We apply different methods, such as interviews and observations within the organization we are collaborating with. In some cases, we also conduct text analysis and analyze documents such as policies and information on websites, *et cetera* (Isaksson et al., 2017).

Based on the research process and findings in the first phase, we identify power relations and constraining norms within the organization in the second phase (analyzing). In most cases, identified norms are related to social categories such as gender, age, sexuality, disability, class, *et cetera*. However, we sometimes identify norms linked to other types of power relations. In the Androchair project, for example, the power relation between the patient and the doctor/midwife was just as essential to problematize as gender relations and gender needs. Regardless of gender, age, sexuality, disability, and class, the patient is in a vulnerable position during gynecological examinations. The patient is almost naked while the doctor is dressed. The doctor/midwife controls the situation while the patient is subordinate and dependent (c.f. Ehrnberger et al., 2017).

In the third phase (visualizing), these norms are visualized through norm-critical design objects. The objects are then presented and discussed with the organization’s members in the fourth phase (discussion and reflection). After these reflexive and awareness-raising activities, the gained insights are translated into new efforts within the organization’s gender equality and diversity work (Isaksson et al., 2017).

In the following section, two of the norm-critical design objects developed in previous projects are exemplified and presented.

### 3.1 The Androchair

The Androchair is an examination chair for men with a design based on women’s experiences with the gynecological chair. In the project that resulted in the Androchair, interviews were conducted with women, midwives, gynecologists, men, andrologists, and urologists, among others. The name “Andro” is linked to andrology—the study of men, which is not as well-known as gynecology. A significant observation made in the project was the lack of routine checks for men regarding reproductive health. It is also not as apparent for men as it is for women where to turn when experiencing discomfort or concerns related to the genitals.

Women interviewed described the gynecological chair as high, unstable, cold, and exposing. Midwives and gynecologists also questioned the design and function of the chair. For example, a midwife expressed during one of the interviews that she usually tells women: “You should lie so that you almost fall out, then you are lying perfectly.” To recreate that feeling, the Androchair was equipped with a tilting function that causes the chair to tilt forward after the patient has settled in. The construction, along with the choice of materials, was made to reflect words such as high, unstable, and cold—which were the words that women raised in the interviews to describe their experiences. Even though the gynecological chair and examination situation were described as somewhat uncomfortable, many argued that it is just something that “is what it is” and something that just “has to be done.” The chair and the situation were normalized. Similar experiences have also been reflected in previous research. Studies have demonstrated that women find the situation and gynecological examination humiliating and exposing, resulting in fears about the visit. This can have significant health consequences. Therefore, a central question raised during the project was why there had been so little development in this area despite women highlighting their negative experiences for several decades. A follow-up question was whether this would have been accepted if the patient group had been men. Another question raised was why andrology does not have as prominent a role as gynecology. In sum, the research group concluded that there were two unmet needs likely associated with normative beliefs about men and women (Ehrnberger, 2017).

**Figure 1:**
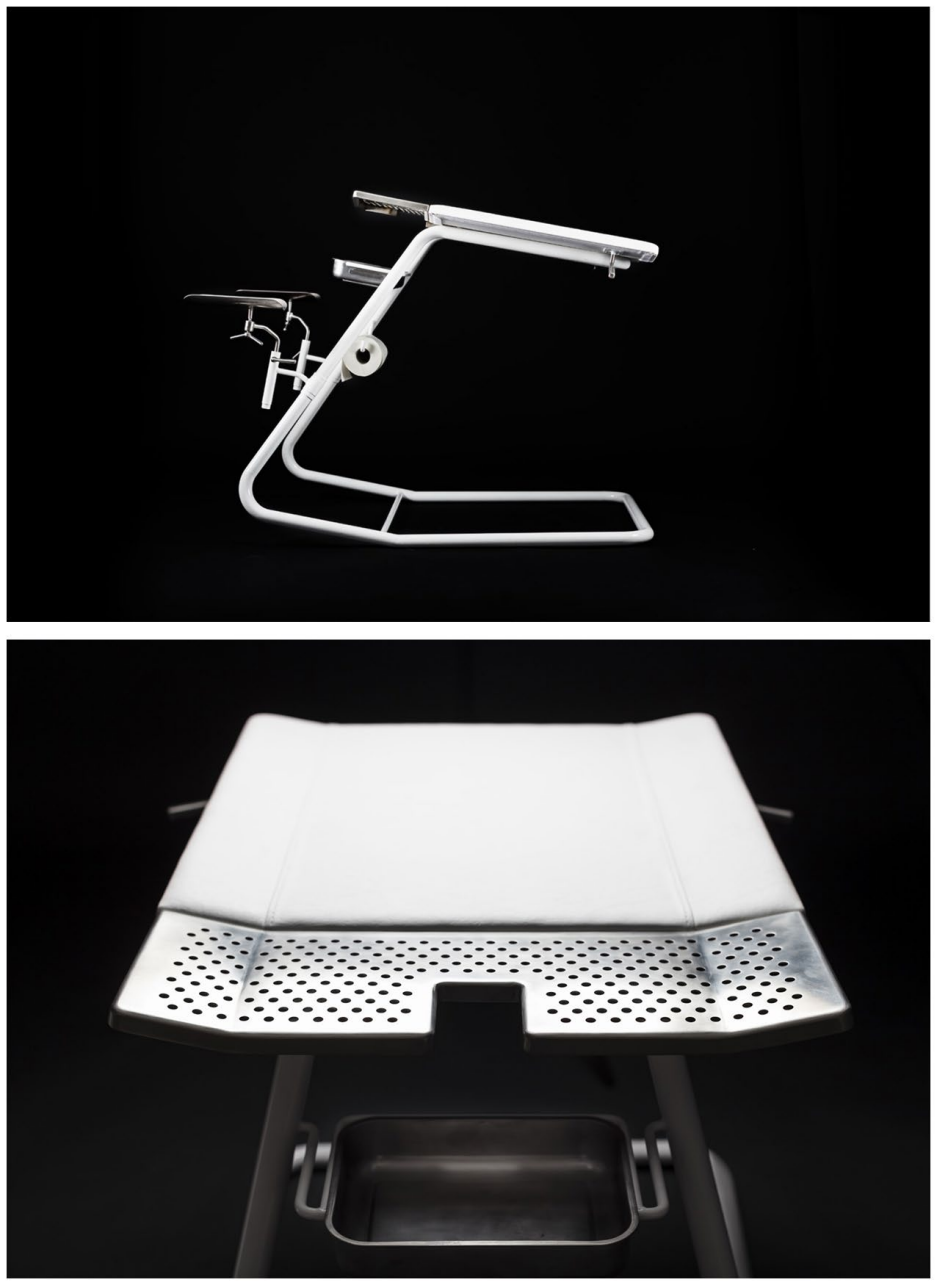
The Androchair. Photo: Anders Andersson.

The Androchair has received attention in media and on social media. As a norm-critical design object, it has been perceived as provocative. Probably, as a consequence of power issues being visualized and questioning which needs are prioritized over others. The Androchair highlights men’s reproductive health and raises why andrology is less evident than gynecology. At the same time, it visualizes women’s experiences and sheds light on the issues surrounding the gynecological chair and gynecological examinations from a (subordinated) patient perspective (Figure 1).

### 3.2 The care song radio

In our project within the female-dominated elderly care, norm-critical design concepts were developed to shed light on how innovation, including technical and digital development, is masculinely coded. This hinders progress and necessary knowledge exchange within female-dominated elderly care, as women perceive that their ideas and experiences are not valued. “Real innovations” are understood as something that should be digital and technical. The project revealed that women in elderly care, daily and innovatively, address challenging situations to provide care for older people. However, today’s prevailing discourse, emphasizing the need for more technical and digital innovations in the welfare sector, results in the invisibility of women’s crucial work, ideas, innovations, and experiences within elderly care (Andersson et al., 2019).

A caregiver who had worked in elderly care for several years shared during an interview how she usually sings a special to an older woman with dementia to ease her anxiety. The song enabled the home care staff to carry out their work, which otherwise would have been almost impossible. The older woman became calm when she heard the song, and the home care staff could help her with toilet visits, showers, and medications. This is an example of how the staff could handle a challenging situation that otherwise hindered their ability to carry out their work. The caregiver knew this song was special and meant a lot to the older woman. Other songs did not have a similar effect, meaning they could not calm the anxiety. This indicates that understanding the individual’s needs is crucial to carrying out caregiving work. However, the caregiver referred to this “solution” as “nothing special.” We also found similar examples where female caregivers downplayed their experiences and essential work. That women do this and make their activities and work invisible was interpreted as a consequence of the solid technical and digital discourse surrounding the welfare sector—a discourse that is strongly masculine-coded. Knowledge gained through experience and intuition, commonly found in elderly care, runs the risk of being overshadowed by technological and digital expertise. Care-based knowledge, experiences, and solutions that cannot be translated into technical/digital products and services are not considered valuable and essential (Isaksson et al., 2018).

The complexity in the caregivers’ story was translated into the “Care Song Radio.” This is a device that sings one song at a time. The front of the radio is simple and directed toward the user. However, the back has many different controls and buttons that set up all the underlying knowledge needed to determine which song suits which person and on what occasion. These settings include information about gender, class, age, cultural identity, diagnosis, weather conditions, prescribed medications, and the current care situation and mood. This information may influence the appropriate genre and song for that specific person at that particular moment. The radio illustrates how such a simple act as singing a song can become very complex when translated into a technical solution. As such, it demonstrates a critique of the technological discourse, and the physical product embodies the underlying knowledge that determines the song. It highlights how crucial the interaction and understanding between the user and caregiver are—caring is complex and not just “natural,” as history has told us (c.f. Andersson et al., 2019).

**Figure 2:**
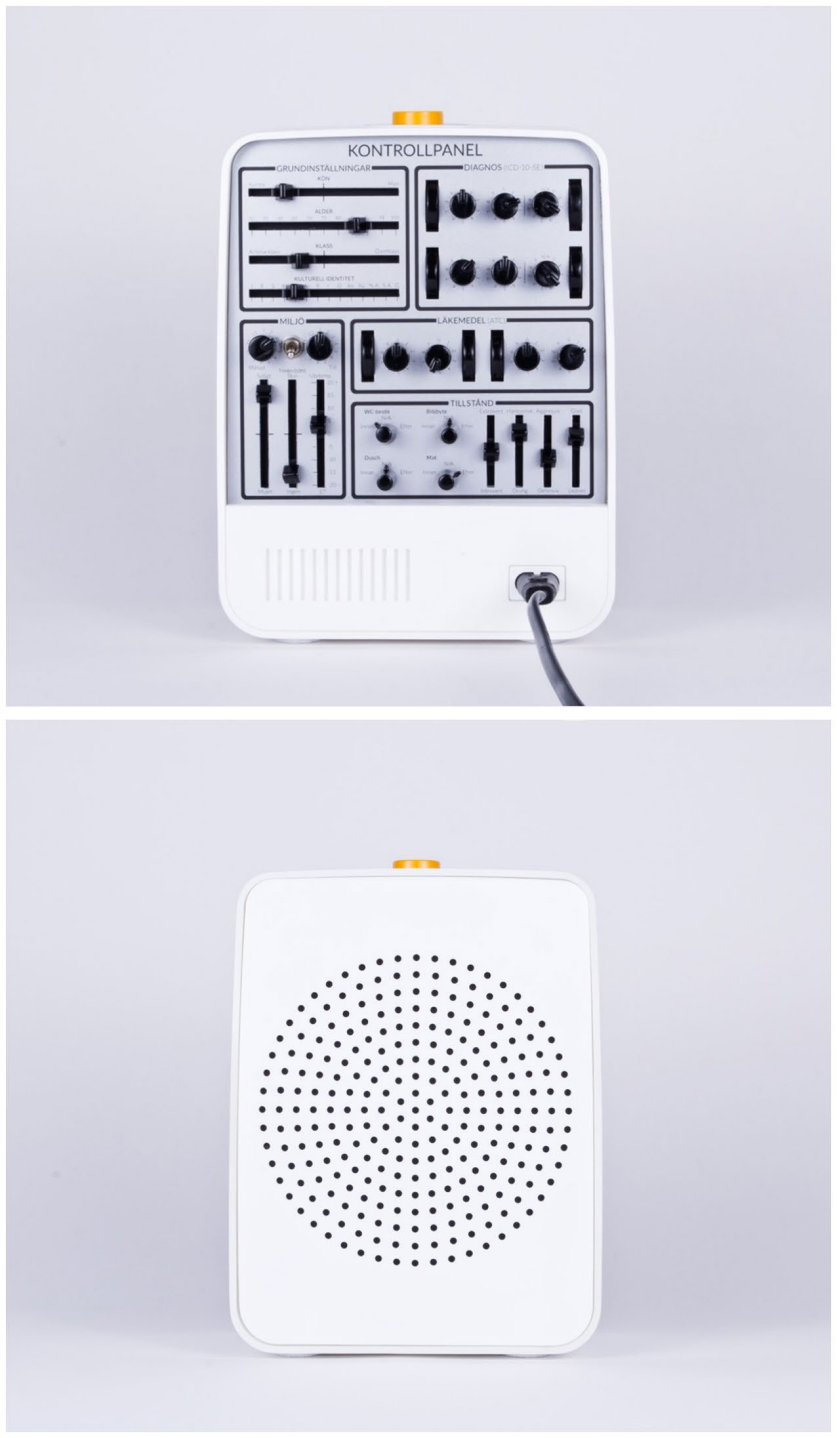
The care song radio—back and front. Photo: David Molander.

In sum, the design object suggests that the depth of knowledge acquired through human caregiving experiences risks being overshadowed by the implementation of technological solutions. Since elderly care is a female-dominated sector, the care song radio emphasizes that female caregivers’ experiences tend to be seen as “natural” and—as the caregiver herself told us—“nothing special” (Figure 2).

## 4 Bringing the Androchair and the care song radio from a research context to the classroom

The two objects evoke various thoughts and reflections, but what unites them is that they result from women’s often neglected experiences. I have introduced the objects to students in sociology courses that concern gender, gender relations, and regimes of inequality. They have also been presented in lectures and seminars related to gender and work, as well as more theoretical moments where Institutional Ethnography of Smith (1987, 2005), often echoed as a method of inquiry beginning from women’s experiences of being locked out from many areas of life and society (*cf.* Pilerot, 2023), are in the center. When introducing two logics of the gender system of Hirdman (1988)—dichotomy (which stipulates that male and female concerns and interests are to be kept separate) and hierarchy (which stipulates that male values are the norm)—the two norm-critical design objects have been presented and discussed in relation to the theory.

The care song radio has also been presented in sessions related to gender and technology. In contrast, the Androchair has been presented and discussed in lectures and seminars on gender and health. Students have always been able to see and feel the objects. I have explained that they are the result of a research method inspired by research through design (Frayling, 1993) and that they are intended to evoke questions, discussions, and reflections. I have also presented their background and research contexts. No more information has been provided to the students.

Overall, students have expressed very positive reactions to gathering around objects. The objects provide a springboard for conversations and, according to what the students raised, helped make the abstract (theories and concepts) more tangible. I have yet to conduct more systematic follow-ups on how the objects have affected students’ learning and also an interview study to capture the students’ point of view. This is necessary to draw more far-reaching conclusions. However, in oral follow-ups, students have also reported that gender issues are perceived as more interesting when they are not only presented in articles, book chapters, theories, and numbers. The objects seem to encourage curiosity among students, and the seminar discussions have become more thorough and diverse, also including intersectional reflections and approaches (c.f. Crenshaw, 1991). The Androchair, for instance, has been discussed in terms of how the gynecological examination can be perceived as more challenging for certain groups than others. For a person who, for example, has a disability (and perhaps has trouble getting onto the examination chair) and identifies as non-binary, the examination can feel even more exposed than for other groups. The care song radio (with its complex back) has raised issues from an intersectional perspective, encouraging discussions about recognizing how various social categories such as gender, class, sexual orientation, age, and disability intersect to create unique experiences in healthcare and social care settings. Further, the object has evoked concerns among the students about how a lack of cultural competency among healthcare and social care providers may lead to misdiagnosis, misunderstandings, and inappropriate treatments. These kinds of reflections emerged from questions such as: What kind of song does an older person who does not speak the dominant language and represents a cultural background other than the staff prefer? How can the staff know what song to sing when the older person struggles to communicate her/his needs due to language barriers and/or disabilities?

## 5 Discussion—the potential of incorporating norm-critical design objects as a pedagogical tool in sociology courses

As mentioned earlier, there is a growing interest in norm-critical design, which means research increasingly yields results (or analytical units) that take on a physical form. Consequently, research findings can be integrated into education through scientific articles, books, reports, *and* tangible norm-critical objects. Further, there is an emerging trend among sociologists engaging with design research methods, which Lupton (2018) calls “design sociology.” Lupton (2018) argues that design research methods are relevant to sociological research, mainly applied research that works toward social change. Previous sociological projects that have adopted a design research approach result in prototypes and objects. However, using design methods in sociology can also, according to Lupton (2018), inspire creative thinking and be a playful and enjoyable way for the public to engage in social research. Hence, design sociology can potentially elicit ideas and reflections that would not arise from more traditional methods within sociology.

Based on my personal insights and experiences, there seems to be a similar potential in integrating design and design objects into sociology courses. Using norm-critical design objects as a pedagogical tool may be beneficial to enhance student creativity and engagement and thus overcome challenges reported in previous research, such as resistance to sociological approaches to gender. Sociology has traditionally been interested in power relations and social orders. As argued by Lupton (2018), design and design methods can contribute with tools for social critique and the identification of social inequalities and power differentials. During my lectures and seminars where I presented the norm critical design objects, I noticed that students, to a greater extent than before, could reflect on intersectional aspects and also identify additional examples of social inequalities beyond those addressed during the lectures. A careful and humble interpretation is that the objects can support students’ ability to develop a reflective and problematizing approach. This observation, however, may not only be attributed to the objects *per se*. Each object resulted from a norm-critical design approach. Thus, it could be argued that the norm-critical lens contributed to the students’ intersectional and critical elaborations on the objects rather than the object itself. Perhaps it also matters that norm-critical design objects in teaching differ from more traditional teaching methods. Like other non-traditional teaching methods such as horror, TikTok, and digital storytelling, the norm-critical design objects seem to have the potential to make course content and concepts more accessible to grasp than if they had only had the course literature available.

In sum, this article suggests that incorporating norm-critical design objects into sociology courses expands the repertoire of pedagogical tools that may enhance student learning and engagement. As of this writing, I am developing a study focusing on the students’ voices and how they perceive norm-critical design objects as pedagogical tools in sociology courses addressing gender and social inequality. Therefore, the aim of this article has been to present how I have utilized norm-critical design objects as a pedagogical tool, and to encourage a broader sociological discussion about the potential of using norm-critical design objects in sociology courses. Consequently, I would like to conclude the article with the following questions: Are there more examples of norm-critical or critical design objects integrated into sociology courses addressing gender issues, other social categories, and/or social inequalities? What do these look like, how do the students perceive them, and how do they influence students’ learning and engagement? Are there related research examples, such as visual sociology, anthropology, ethnography, *et cetera*, where design objects resulting from research have been integrated into higher education courses? What can we learn from these studies?

## Data availability statement

The original contributions presented in the study are included in the article/supplementary material, further inquiries can be directed to the corresponding author.

## Author contributions

AI: Writing – original draft, Writing – review & editing.

## References

[ref9003] Andersson C. Mazé R. Isaksson A. (2019). “Who cares about those who care?: Design and technologies of power in Swedish elder care,” in WHO CARES? 8th biannual Nordic Design Research Society (Nordes) conference. Espoo, Finland: Aalto University, 2019.

[ref1] Bardzell J. Bardzell S. (2013). “What is critical about critical design?” in *Proceedings of the SIGCHI Conference on Human Factors in Computing Systems (CHI '13)*, Paris. ACM, pp. 3297–3306.

[ref2] Bardzell S. Bardzell J. Forlizzi J. Zimmerman J. Antanitis J. (2012). “Critical design and critical theory: The challenge of designing for provocation” in *Proceedings of the Designing Interactive Systems Conference (DIS '10)*, Newcastle, UK. ACM, pp. 288–297.

[ref3] Berkowitz D. Manohar N. N. Tinkler J. E. (2010). Walk like a man, talk like a woman: teaching the social construction of gender. Teach. Sociol. 38, 132–143. doi: 10.1177/0092055X10364015

[ref4] Binder T. De Michelis G. Ehn P. Jacucci G. Linde P. Wagner I. (2011). Design Things. Cambridge, MA: MIT Press.

[ref9002] Börjesson E Isaksson A Ilstedt S Ehrnberger K. (2016) “Visualizing gender–Norm-critical design and innovation,” in Research handbook on gender and innovation. eds. Alsos G. A. Hytti U. Ljunggren E.. (Cheltenham: Edward Elgar Publishing), 252–274.

[ref5] Costanza-Chock S. (2020). Design Justice: Community-Led Practices to Build the Worlds We Need. The MIT Press.

[ref6] Crenshaw K. (1991). Mapping the margins: intersectionality, identity politics, and violence against women of color. Stanford Law Rev. 43, 1241–1299. doi: 10.2307/1229039

[ref7] Denz S. Eggink W. (2019). “Queer-sensible designing: challenging normative gender through an industrial design practice” in *Academy for Design Innovation Management Conference 2019: Research perspectives in the era of Transformations*. London, UK. 1–20.

[ref8] Dunne A. Raby F. (2013). Speculative Everything: Design, Fiction, and Social Dreaming. Cambridge, MA: The MIT Press.

[ref9] Ehrnberger K. (2017). Tillblivelser. En trasslig berättelse om design som normkritisk praktik. KTH PhD dissertation. KTH, Stockholm.

[ref10] Ehrnberger K. Räsänen M. Börjesson E. Hertz A.-C. Sundbom C. (2017). The Androchair: performing Gynaecology through the practice of gender critical design. Des. J. 20, 181–198. doi: 10.1080/14606925.2016.1261510

[ref11] Ehrnberger K. Räsänen M. Ilstedt S. (2012). Visualising gender norms in design: meet the mega hurricane mixer and the drill dolphia. Int. J. Des. 6, 85–98. doi: 10.7551/mitpress/12255.001.0001

[ref12] Frayling C. (1993). Research in Art and Design, RCA Research Papers, 1: 1. London: Royal College of Art.

[ref13] Fuenfschilling L. Paxling L. Perez E. V. (2022). Norm-critical innovation as a way forward for responsible innovation? Evidence from a Swedish innovation policy program. J. Respons. Innov. 9, 371–397. doi: 10.1080/23299460.2022.2112817

[ref14] Gaver W. Bowers J. Boehner K. Boucher A. Cameron D. W. T. Hauenstein M.. (2013). “Indoor weather stations: Investigating a ludic approach to environmental HCI through batch prototyping” in *Proceedings of the SIGCHI Conference on Human Factors in Computing Systems (CHI '13)*, Paris. ACM, pp. 3451–3460.

[ref15] González-González C.S. García-Holgado A. Garcia-Peñalvo F. J. (2020). “Strategies to introduce gender perspective in engineering studies: a proposal based on self-diagnosis” in *2020 IEEE Global Engineering Education Conference (EDUCON), Porto, Portugal*, pp. 1884–1890.

[ref16] Hartless J. (2021). Horror as a pedagogical tool for teaching sexualities. Teach. Sociol. 49, 233–244. doi: 10.1177/0092055X211022458

[ref17] Heijstra T. M. Pétursdóttir G. M. (2023). Capturing dis/comfort and navigating transformation in the gender studies classroom. Teach. Sociol. 51, 349–361. doi: 10.1177/0092055X221149441

[ref18] Hirdman Y. (1988). Genussystemet—Teoretiska funderingar kring kvinnors sociala underordning (Gender system—Theoretical reflections on women’s social subordination). Maktutredningen, p. 23. Institute for Working Life, Stockholm.

[ref19] Horwath I. Diabl C. (2019). Liberating or indoctrinating? Surveying students’ perceptions of a Women’s and gender studies requirement. Gend. Educ. 32, 1109–1126. doi: 10.1080/09540253.2019.1608355

[ref9001] Isaksson A. Börjesson E. Gunn M. Andersson C. Ehrnberger K. (2017). Norm critical design and ethnography: Possibilities, objectives and stakeholders. Sociol. Res. Online 22, 232–252. doi: 10.1177/1360780417743168

[ref01] Isaksson A. Börjesson E. Andersson C. Ehrnberger K. (2018). Samverkansformer: Nya vägar för humaniora och samhällsvetenskap. eds. Berg M. Fors V. Willim R. (Lund: Studentlitteratur AB), 153–173.

[ref20] Kaygan H. Kaygan P. Demir Ö. (2019). A pen that ‘looks like a CEO in a business suit’: gendering the fountain pen. J. Gend. Stud. 28, 86–96. doi: 10.1080/09589236.2017.1409105

[ref21] Lampe N. M. (2023). Teaching with TikTok in online sociology of sex and gender courses. Teach. Sociol. 51, 323–335. doi: 10.1177/0092055X231159091

[ref22] Liu W. (2024). The role of design in society. Des. J. 27, 385–387. doi: 10.1080/14606925.2024.2353478

[ref23] López-Orozco C. F. López-Caudana E. O. Ponce P. (2022). A systematic mapping literature review of education around sexual and gender diversities. Front. Sociol. 7:946683. doi: 10.3389/fsoc.2022.946683, PMID: 36081574 PMC9445552

[ref24] Lupton D. (2018). Towards design sociology. Sociol. Compass 12:e12546. doi: 10.1111/soc4.12546

[ref25] Malpass M. (2013). Between wit and reason: defining associative, speculative, and critical design in practice. Des. Cult. 5, 333–356. doi: 10.2752/175470813X13705953612200

[ref26] McCabe J. (2013). Making theory relevant: the gender attitude and belief inventory. Teach. Sociol. 41, 282–293. doi: 10.1177/0092055X13480153

[ref27] Michael M. (2016). Notes toward a speculative methodology of everyday life. Qual. Res. 16, 646–660. doi: 10.1177/1468794115626245

[ref28] Miller J. Chamberlin M. (2000). Women are teachers, men are professors: a study of student perceptions. Teach. Sociol. 28, 283–298. doi: 10.2307/1318580

[ref29] Pilerot O. (2023). “Information literacy theorised through institutional ethnography” in Information Literacy Through Theory. eds. Hicks A. Lloyd A. Pilerot O. (UK: Facet Publishing).

[ref30] Sengers P. Boehner K. David S. Kaye J. J. (2005). “Reflective design” in *Proceedings of the 4th Decennial Conference onCritical Computing: Between Sense and Sensibility*, *Aarhus*. ACM, pp. 49–58.

[ref31] Smith D. E. (1987). The Everyday World as Problematic: A Feminist Sociology. Toronto: University of Toronto Press.

[ref32] Smith D. E. (2005). Institutional Ethnography: A Sociology for People. Walnut Creek, CA: AltaMira Press, Rowman & Littlefield Publishers.

[ref9005] Titus J. J. (2010). Engaging Student Resistance to Feminism: “How is this stuff going to make us better teachers?” Gender and Education 12, 21–37. doi: 10.1080/09540250020382

[ref33] Trumpy A. (2023). “Integrating gender, sex, and sexuality throughout the curriculum” in Handbook of Teaching and Learning in Sociology. eds. Cabrera S. Sweet S. (Northampton, MA: Edward Elgar Publishing).

[ref34] Vaughn M. P. Leon D. (2021). The personal is political art: using digital storytelling to teach sociology of sexualities. Teach. Sociol. 49, 245–255. doi: 10.1177/0092055X211022459, PMID: 35811837 PMC9267295

[ref35] Wickström A. Zeiler K. (2021). The performativity of surveys: teenagers’ meaning-making of the 'Health behavior in school-aged children Survey' in Sweden. Child. Soc. 35, 428–444. doi: 10.1111/chso.12425

[ref36] Wilkie A. Michael M. Plummer-Fernandez M. (2015). Speculative method and twitter: bots, energy and three conceptual characters. Sociol. Rev. 63, 79–101. doi: 10.1111/1467-954X.12168

[ref37] Yetiş E. Ö. Bakırlıoğlu Y. (2024). Dis/re-orienting design through norm-critical gender lenses: an educational case in Turkey. Front. Sociol. 9:1341091. doi: 10.3389/fsoc.2024.1341091, PMID: 38606054 PMC11007197

